# Inventory of the Secondary Metabolite Biosynthetic Potential of Members within the Terminal Clade of the *Fusarium solani* Species Complex

**DOI:** 10.3390/jof9080799

**Published:** 2023-07-28

**Authors:** Ambika Pokhrel, Jeffrey J. Coleman

**Affiliations:** 1 Department of Entomology and Plant Pathology, Auburn University, Auburn, AL 36849, USA; azp0091@auburn.edu; 2 The Donald Danforth Plant Science Center, St. Louis, MO 63132, USA

**Keywords:** nonribosomal peptide synthetase (NRPS), polyketide synthase (PKS), terpene synthase/cyclase, siderophore, radicicol, gibberellin

## Abstract

The *Fusarium solani* species complex (FSSC) constitutes at least 77 phylogenetically distinct species including several agriculturally important and clinically relevant opportunistic pathogens. As with other Fusaria, they have been well documented to produce many secondary metabolites—compounds that are not required for the fungus to grow or develop but may be beneficial to the organism. An analysis of ten genomes from fungi within the terminal clade (clade 3) of the FSSC revealed each genome encoded 35 (*F. cucurbitcola*) to 48 (*F. tenucristatum*) secondary metabolite biosynthetic gene clusters (BGCs). A total of seventy-four different BGCs were identified from the ten FSSC genomes including seven polyketide synthases (PKS), thirteen nonribosomal peptide synthetases (NRPS), two terpene synthase BGCs, and a single dimethylallytryptophan synthase (DMATS) BGC conserved in all the genomes. Some of the clusters that were shared included those responsible for producing naphthoquinones such as fusarubins, a red pigmented compound, squalestatin, and the siderophores malonichrome, ferricrocin, and triacetylfusarinine. Eight novel NRPS and five novel PKS BGCs were identified, while BGCs predicted to produce radicicol, gibberellin, and fusaoctaxin were identified, which have not previously described in members of the FSSC. The diversity of the secondary metabolite repertoire of the FSSC may contribute to the expansive host range of these fungi and their ability to colonize broad habitats.

## 1. Introduction

Many fungi are capable of synthesizing complex compounds with bioactive properties that are not essential for the organism. These compounds, termed secondary metabolites, can have diverse chemical properties and are usually produced by a cluster of genes within the fungal genome [1]. These biosynthetic gene clusters (BGCs) frequently contain a key gene encoding the enzyme responsible for the synthesis of the “core” or “backbone” unit of the compound and may include accessory genes that are responsible for further modification(s) to the compound. The two most common classes of key genes found in BGCs encode either polyketide synthases (PKSs) or nonribosomal peptide synthetases (NRPSs). PKS enzymes generate a compound from linking ketides into a chain and are further divided into non-reducing and reducing PKS proteins, which are responsible for generating aromatic and fatty acid-like molecules, respectively. NRPSs catalyze the production of peptide-based compounds from individual amino acids. Other key enzymes in BGCs responsible for secondary metabolite biosynthesis include terpene synthase/cyclases, which produce compounds with the formula (C_5_H_8_)_n_, dimethylallytryptophan synthases (DMATS) producing derivatives of tryptophan, as well as BGCs encoding a phosphoenolpyruvate phosphomutase responsible for producing phosphonate compounds. The most prolific fungal producers of secondary metabolites are members of the Ascomycota, which produce well-known secondary metabolites such as the medically relevant compounds penicillin and lovastatin and the mycotoxins aflatoxin and deoxynivalenol (also known as vomitoxin).

Members of the ascomycete genus *Fusarium* produce a myriad of secondary metabolites including several mycotoxins. As a genus, *Fusarium* is composed of hundreds of species separated into at least 23 phylogenetically distinct species complexes [2,3]. While the genus is estimated to have originated ~91 million years ago during the Cretaceous period [4], the group referred to as the *F. solani* species complex (FSSC) diverged from the rest of the Fusaria ~67 million years ago [4]. Currently, the FSSC is divided into at least 77 phylogenetically distinct species and are found worldwide [3,5]. This species complex is composed of three clades (clades 1–3) where the terminal clade 3 is the largest with at least 61 distinct species and is composed of many agriculturally important species as well as those associated with clinical infections [3,5,6,7,8].

Many secondary metabolites have been identified from members of the FSSC. Primarily these have been napthoquinones including fusarubin and a multitude of derivatives and similarly structured compounds (javanicin, solaniol, matricin, bikaverin, trichodermaol, etc.) [9,10]. Other confirmed secondary metabolites include citreoisocoumarin, cyclosporin, gibepyrone, lucilactaene, N-carbenzoxy-L-phenylalaninol, sansalvadmide, YCM1008A, as well as unknown metabolites including a red pigment [10,11]. Of these compounds, only the production of sansalvadmide (*PKS30*) and the red pigment (*PKS35* also referred to as *PKSN*) has been experimentally linked to specific BGCs in isolates from the FSSC [12,13].

Secondary metabolites play an important role in fungal pathogenicity on plants and animals. Additionally, many secondary metabolites have inhibitory activity against other microbes, and therefore may influence the soil microbiome through their production. As members of the FSSC are soil-borne plant pathogens, evaluation of their secondary metabolite biosynthetic potential is essential to better understand these pathogens. Analysis of ten FSSC genomes revealed BGCs that were shared between all the FSSC genomes while some were unique to a single genome. Despite the abundance of these BGCs in the FSSC, the biosynthetic product of a vast majority of these BGCs remains unknown.

## 2. Materials and Methods

### 2.1. Genomic Data of FSSC Isolates

The relevant information of the FSSC genomes included in this study are listed in Table 1. The genome sequence and annotation of seven FSSC isolates were obtained from Mycocosm at the Joint Genome Institute (JGI) and three genomes were obtained from the National Center for Biotechnology Information (NCBI). A revised version of the *F. vanettenii* 77-13-4 genome assembly and annotation was used in this study resulting in a discrepancy in the protein IDs of the PKS and NRPS proteins provided in Tables S2–S5 and protein IDs reported in previous studies [14]. The predicted number of BGCs in the newer version of the *F. vanettenii* 77-13-4 genome was different (total 39) compared to the previous version (total 36).

In addition to the genome data listed in Table 1, the PKS and NRPS reference protein sequences from other *Fusarium* spp. and fungal species were obtained from GeneBank and included in the phylogenetic analyses for comparison (Tables S2 and S3).

### 2.2. Species Phylogeny of FSSC Isolates

The gene sequences of translation elongation factor (*TEF1*) and the subunits of RNA-dependent polymerase (*RPB1* and *RPB2*) for all the FSSC isolates were identified using BLAST+ [18]. A multiple gene sequence alignment was conducted in MEGA11 [19], and the gaps and non-conserved regions were trimmed using Gblocks [20]. Model selection and a maximum likelihood phylogeny with 1000 bootstrap replicates were constructed using MEGA11 [19].

### 2.3. Prediction of Secondary Metabolite Gene Clusters and Phylogenetic Analysis of PKS and NRPS Proteins

The secondary metabolite gene clusters in the FSSC genomes were predicted using the fungal version of antiSMASH 6.0 using the options KnownClusterBlast, ClusterBlast, and SubClusterBlast [21]. The number of biosynthetic gene clusters (BGCs) for each genome was calculated and the PKS and NRPS protein sequences from these predicted clusters were extracted. The PKS and NRPS proteins from both the previous and revised versions of the *F. vanettenii* 77-13-4 genomes were included in the analysis to compare the differences in annotation. The PKS and NRPS proteins were classified into various groups separately using a phylogeny-based approach. Already known PKS and NRPS reference protein sequences from various fungal genomes (Tables S2 and S3) were also included in the analysis to aid in the classification [14,22,23,24]. For the phylogenetic analysis, PKS and NRPS protein sequences from all FSSC isolates and reference protein sequences were aligned using MAFFT [25]. All the gaps and non-conserved regions were removed from the alignment using BMGE [26]. The best protein model selection was conducted using the program ProtTest 3.4 [27], and maximum likelihood phylogeny trees with 1000 bootstrap replicates were generated using RAxML 8.0 [28]. Visualization of the phylogenetic trees was completed in iTOL v6 [29]. The PKS and NRPS clades were classified based on the reference proteins.

### 2.4. Similarity Network and Phylogenetic Analysis of the Terpene Synthase/Cyclase, DMATS, and Phosphoenolpyruvate Phosphomutase

The similarity network analysis and the diversity of the BGCs responsible for the production of terpenes, dimethylallytryptophan derivatives, and phosphonate compounds were explored using the program BiG-SCAPE [30]. BiG-SCAPE is a genome mining tool that facilitates the fast and interactive analysis of BGCs from multiple genomes, develops similarity networks, and classifies the clusters into various gene cluster families (GCFs). All the reference BGCs from the MIBiG database [31] were included in the analysis to identify the similarities with known natural products.

In addition to developing the similarity networks of BGCs and classifying various GCFs, BiG-SCAPE elucidates the phylogenetic relationship within these families using a multi-locus phylogeny approach. The phylogenetic analysis of the terpenes, dimethylallytryptophan derivatives, and phosphonate-producing BGCs was accomplished using the program BiG-SCAPE [30], which also generated the genetic organization figures for each of these BGCs. The phylogenetic tree files from the BiG-SCAPE analyses were visualized using MEGA [19] and arranged manually.

### 2.5. Analysis of the Genetic Organization of the Conserved and Unique Biosynthetic Gene Clusters with Known Products

The GeneBank files of the conserved BGCs from PKS and NRPS groups, as well as the unique BGCs: radicicol, gibberellin, and fusaoctaxin A, were extracted from the antiSMASH output. If the BGC was similar to any previously characterized cluster as determined by antiSMASH results, the corresponding GeneBank file was obtained from the MIBiG repository of known BGCs [31]. These homologous BGCs were compared and visualized using the program clinker [32], generating the gene cluster comparison figures.

## 3. Results

### 3.1. Phylogenetic Analysis of FSSC Isolates Used in This Study

**Figure 1:**
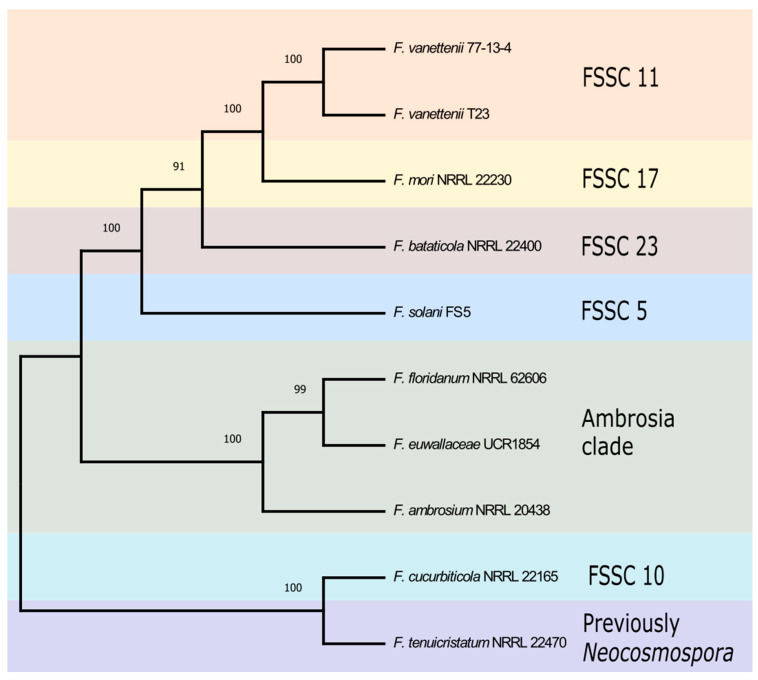
Phylogenetic analysis of ten FSSC isolates analyzed in this study. The maximum-likelihood phylogenetic tree of ten FSSC isolates from the terminal clade was constructed using the coding sequences of *TEF1*, *RPB1*, and *RPB2*. The robustness of the tree was assessed using 1000 bootstrap replicates.

To better understand the evolutionary relationship between the FSSC isolates used in this study, a phylogenetic analysis was generated using three phylogenetically informative loci including the coding genes of *TEF1*, *RPB1*, and *RPB2*. A maximum-likelihood phylogenetic tree using the concatenated three gene sequences allowed for the visualization of the relationship between the ten FSSC genomes (Figure 1). The organization of the FSSC isolates in the phylogenetic tree was in agreement with previous studies [3], placing the three FSSC isolates (*F. floridanum*, *F. euwallaceae*, and *F. ambrosium*) in the ambrosia *Fusarium* clade.

### 3.2. Identification of Secondary Metabolite Biosynthetic Clusters

The secondary metabolite biosynthetic gene clusters (BGCs) for all ten FSSC genomes were identified using the fungal version of antiSMASH 6.0 [21]. The antiSMASH analysis revealed the FSSC genomes encoded an average of 41 BGCs, with the least in *F. cucurbitcola* with 35 and the most in *F. tenuicristatum* with 48 (Table 2). The predicted number of BGCs in the two genomes of *F. vanettenii*, 77-13-4 and T23, were 39 and 42, respectively, indicating that diversity in the secondary metabolite potential exists within the same species (Table 2). The revised version of the *F. vanettenii* 77-13-4 genome identified 39 BGCs, 3 more than in the prior version. The FSSC isolates were predicted to produce PKS, NRPS, terpene synthase/cyclase, DMATS, phosphonate biosynthetic, and hybrid BGCs. The BGCs containing a PKS or NRPS gene as the main biosynthetic core gene were the most abundant gene clusters followed by terpene synthase/cyclase- containing clusters. Among these BGCs, only *F. bataticola* NRRL 22400 was predicted to encode a PKS–terpene hybrid cluster. Two ambrosia *Fusarium* clade genomes, *F. floridanum* NRRL 62606 and *F. euwallaceae* UCR 1854, were capable of producing an NRPS–terpene hybrid cluster and the *F. bataticola* and *F. solani* FS5 genomes were predicted to carry an NRPS–DMATS hybrid cluster.

Of the putative BGCs, natural products for only a few were able to be predicted using the KnownClusterBlast function in antiSMASH (Table S1). The natural products squalestatin, a red pigment, and fusarubins/oxyjavanicin as well as several potential siderophores including fusarinine, ferricrocin, malonichrome, and metachelin, were predicted to be produced by all ten FSSC isolates analyzed in the study. Sansalvamide (NRPS30) and clavaric acid were predicted to be produced by all the FSSC isolates with the exception of those included in the ambrosia *Fusarium* clade (Table S1). An NRPS–PKS hybrid BGC in *F. solani* FS5 and *F. tenuicristatum* was predicted to be responsible for the synthesis of cyclosporin, a secondary metabolite that has previously been described from members of the FSSC [10,33].

### 3.3. Comparison of the Polyketide Synthase (PKS)-Containing Clusters

**Figure 2:**
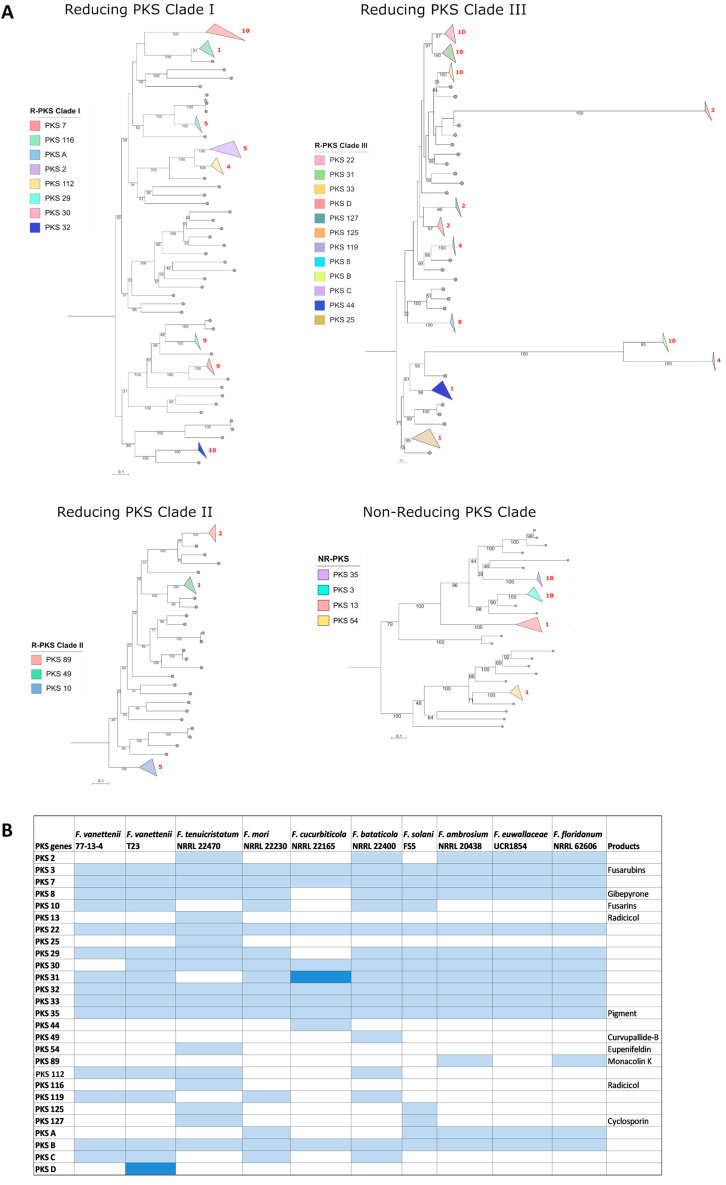
Overview of the distribution of PKS-encoding genes across FSSC genomes. (**A**) Maximum likelihood phylogenetic tree of 290 PKS proteins from FSSC genomes and reference PKS proteins from *Fusarium* and various other fungal species. The robustness of the tree was assessed using 1000 bootstrap replicates. The clades with PKS proteins from 10 FSSC genomes are highlighted in various colors in the phylogenetic tree. (**B**) The detailed distribution of PKS genes in FSSC genomes. Known products of the PKS-containing BGC are also provided. Light blue color indicates the presence of one core biosynthetic gene and the dark blue color indicates the presence of two or more core biosynthetic genes. Previously undescribed PKS genes are indicated with letters, i.e., PKS A-D.

PKS reference proteins from *Fusarium* and other fungal species (Table S2; [14,22,23,24]) and the PKS proteins (including those in hybrid PKS BGCs) from our FSSC study group (Table S4) were included in the analysis for a total of 290 PKS amino acid sequences. The phylogenetic analysis of the PKS proteins revealed 27 different groups of PKS orthologues in the FSSC genomes used in this study (Figure 2 and Figure S1, Table S4), and were represented by 23 previously known PKS proteins in other *Fusarium* spp. and 4 that were previously undescribed (PKSA-D; Figure 2B). BGCs containing *PKS3*, *PKS7*, *PKS22*, *PKS32*, *PKS33*, *PKS35*, and the previously undescribed *PKSB* were conserved in all the FSSC genomes included in this study. Among the 27 PKS BGC groups, only the products of 10 were able to be predicted (Figure 2) based on antiSMASH results and previous studies [11,14]. The secondary metabolites fusarubins (PKS3), gibepyrone (PKS8), and the red pigment (PKS35) are the currently known conserved secondary metabolites from the FSSC [11]. While the core biosynthetic gene *PKS3* is moderately conserved across the ten FSSC isolates in this study, an analysis of the genetic organization of the entire *PKS3* BGC responsible for fusarubin/oxyjavanicin synthesis revealed that the accessory genes of the ambrosia FSSC genomes were different compared to the other FSSC isolates (Figure S2). Previously, the gene encoding a PKS responsible for the production of a red pigment (termed *PKSN/PKS35*) was characterized [13], and the genetic organization of the BGC encoding *PKS35* was conserved across all the FSSC isolates (Figure S3).

### 3.4. Comparison of the Nonribosomal Peptide Synthetase (NRPS)-Containing Clusters

**Figure 3:**
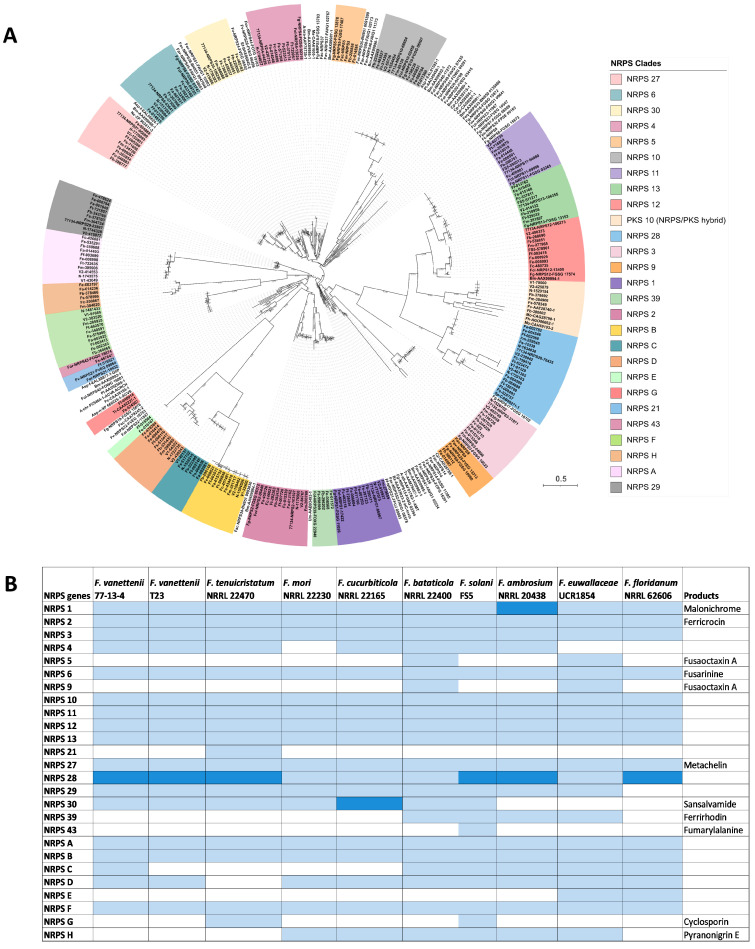
Overview of the distribution of NRPS-encoding genes across FSSC genomes. (**A**) Maximum likelihood phylogenetic tree of 332 NRPS proteins from FSSC genomes and reference NRPS proteins from *Fusarium* and various other fungal species. The robustness of the tree was assessed using 1000 bootstrap replicates. The clades with NRPS proteins from 10 FSSC genomes are highlighted in various colors in the phylogenetic tree. (**B**) The detailed distribution of NRPS genes in FSSC genomes. Known products of the NRPS-containing BGC are also provided. Light blue color indicates the presence of one core biosynthetic gene and the dark blue color indicates the presence of two or more core biosynthetic genes. Previously undescribed NRPS genes are indicated with letters, i.e., NRPS A-H.

A phylogenetic analysis of 332 NRPS proteins was conducted representing NRPS proteins identified from the antiSMASH analysis of the FSSC genomes (including the NRPSs in hybrid BGCs) and using NRPS reference proteins from other *Fusarium* and fungal species (Table S3) [23,24]. Twenty-six different clades of NRPS proteins were distinguished (Figure 3 and Table S5), of which eighteen were previously identified in other *Fusarium* spp. and eight (NRPSA-H) were previously undescribed NRPS proteins found in the FSSC genomes.

Of the 26 NRPSs in the FSSC genomes, 13 were conserved in all 10 genomes (*NRPS1*, *NRPS2*, *NRPS3*, *NRPS6*, *NRPS10*, *NRPS11*, *NRPS12*, *NRPS13*, *NRPS27*, *NRPS28*, *NRPSA*, *NRPSB*, and *NRPSF*; Figure 3). Conversely, *NRPS21* and *NRPS43* were found in only a single FSSC genome, *F. tenuicristatum* NRRL 22470 and *F. solani* FS5, respectively. Many of the conserved NRPS BGCs were predicted to be responsible for the biosynthesis of the siderophores malonichrome (*NRPS1*), ferricrocin (*NRPS2*), triacelyfusarinine (*NRPS6*), and metachelin (*NRPS27*) (Figures S4–S7). The only NRPS in the FSSC that has been experimentally confirmed is NRPS30, responsible for sansalvamide biosynthesis [12], and it was present in all FSSC genomes included in this study except for those in the ambrosia *Fusarium* clade (Figure 3). *NRPS43* is predicted to contribute to the biosynthesis of fumarylalanine, and a single BGC containing this NRPS was identified in the genome of *F. solani* FS5 (Figure 3). Two NRPS-encoding genes, *NRPS5* and *NRPS9*, were found to reside in a single BGC in the genomes of *F. bataticola* and *F. euwallaceae* and homologues of these NRPS genes have been characterized in *F. graminearum* and are known to produce the octapeptide fusaoctaxin A [34].

### 3.5. Comparison of the Terpene Synthase/Cyclase, DMATS, and Phosphonate-Producing BGCs

**Figure 4:**
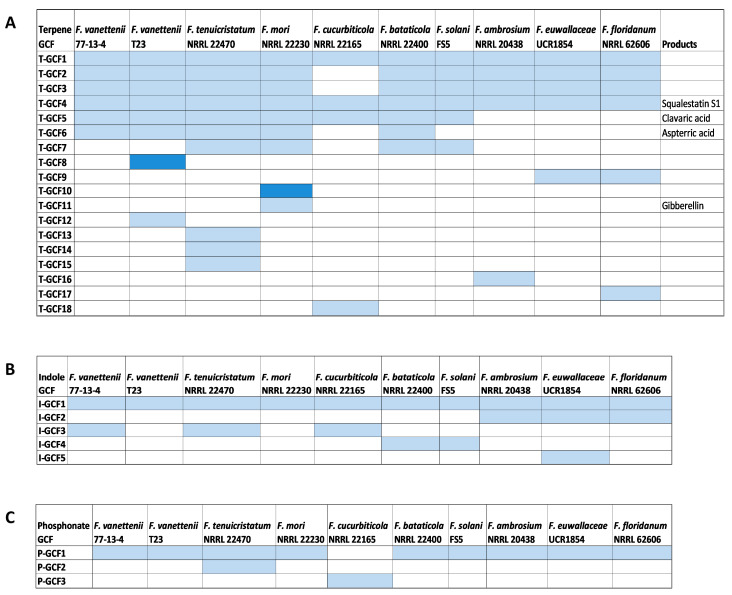
Distribution of terpene synthases/cyclases, DMATS, and BGCs responsible for the synthesis of phosphonate compounds across the FSSC genomes. Similarity network analysis of the terpene synthases/cyclases, DMATS, and phosphonate-generating BGCs was conducted to identify their distribution and conservation across the FSSC genomes. The product name is indicated alongside the gene cluster family (GCF) if predicted or known. GCF grouping of (**A**) terpene synthases/cyclases (T-GCF), (**B**) DMATS (I-GCF), and (**C**) phosphonate-producing BGCs (P-GCF) are shown across the FSSC genomes. Light blue color indicates the presence of one core biosynthetic gene and the dark blue color indicates the presence of two or more core biosynthetic genes.

A total of 98 BGCs responsible for the production of terpenes, dimethylallytryptophan derivatives, and phosphonate compounds were predicted from the 10 FSSC genomes using antiSMASH and were further analyzed using BiG-SCAPE [30]. Of all these BGCs within these biosynthetic groups, only two terpene- synthase/cyclase-containing clusters and a single DMATS were conserved between all ten FSSC genomes (Figure 4). The multi-locus phylogenetic analysis of these GCFs (Figures S8–S10) revealed that their phylogenetic grouping is mostly in accordance with their species phylogeny (Figure 1).

**Figure 5:**
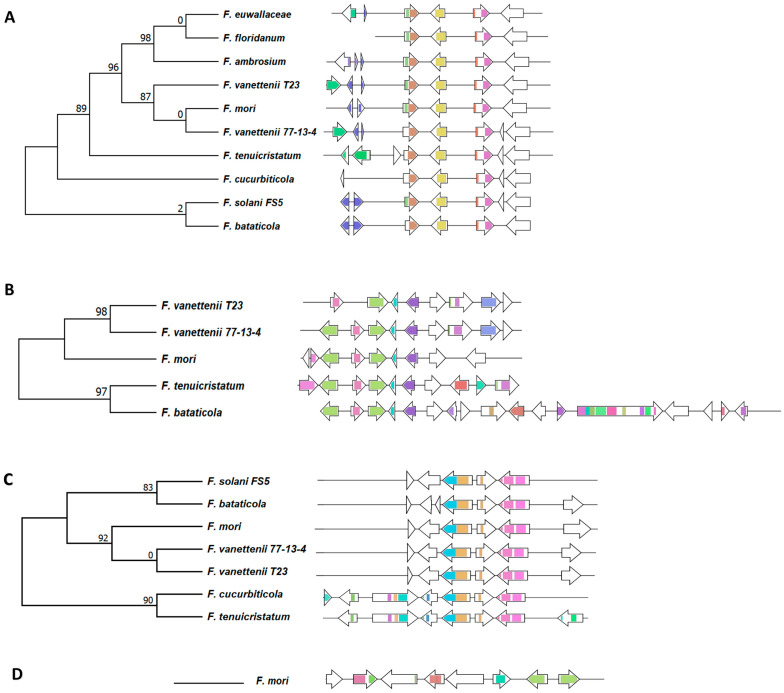
Analysis of the terpene GCFs with predicted or known products. (**A**) Squalestatin, (**B**) aspterric acid, (**C**) clavaric acid, and (**D**) gibberellin. The multi-locus phylogenetic analysis of the GCFs generated by BiG-SCAPE is shown along with the gene cluster organization within the FSSC genomes. Each arrow represents a different gene, and the color represents various functional domains.

The analysis of 67 terpene-synthase-/cyclase-containing BGCs revealed 18 different terpene synthase/cyclase gene cluster families (T-GCFs), including 10 singletons (Figure 4A). Of these 18 T-GCFs, a product of 5 of these terpene synthase/cyclase-containing clusters was predicted. Squalestatin was predicted to be produced by all ten FSSC isolates; its amino acid similarity and the synteny of the cluster indicates that it was conserved during vertical inheritance throughout the terminal clade of the species complex (Figure 4, Figure 5A and Figure S8). Aspterric acid was predicted to be produced by five FSSC isolates, while a BGC from *F. bataticola* was a hybrid PKS–terpene cluster (Figure 4, Figure 5B and Figure S8). Seven FSSC genomes were predicted by antiSMASH to have the terpene synthase BGC responsible for the production of clavaric acid (T-GCF-6; Figure 4 and Figure 5C).

Nineteen total BGCs containing a DMATS were identified across the ten FSSC genomes, and only one DMATS-encoding BGC was conserved between all FSSC genomes in the analysis (Figure 4B and Figure S9). A single conserved BGC encoding a putative phosphoenolpyruvate phosphomutase was present in all FSSC genomes with the exception of *F. cucurbiticola* NRRL 22165, which had a unique phosphonate-producing BGC (Figure 4C and Figure S10).

### 3.6. Unique and Specific BGCs of Interest

**Figure 6:**
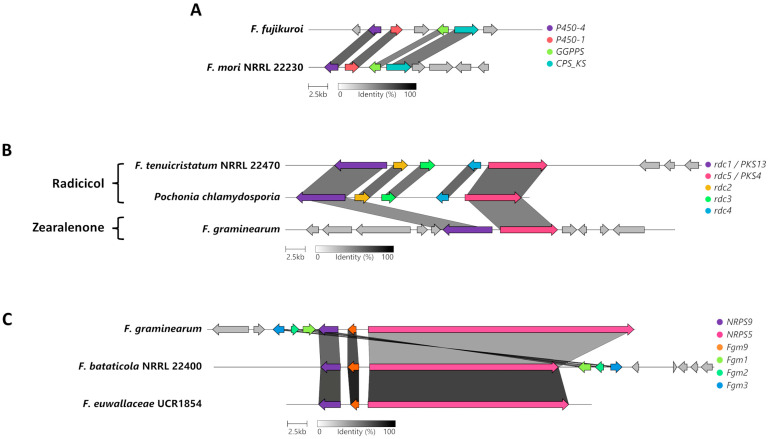
Genetic organization and conservation of the BGCs of interest. Conservation and biosynthesis of (**A**) gibberellin, (**B**) radicicol, and (**C**) fusaoctaxin A. Each color represents a different gene and are named based on the gene names of the originally described or characterized cluster.

Phylogenetic analysis indicated that the genome of *F. mori* carries a BGC that is similar to the BGC responsible for gibberellin biosynthesis in *F. fujikouri* (Figure 5D). Further analysis between these two gibberellin BGCs uncovered four homologous genes and the organization of the cluster was conserved (Figure 6A). The homologue encoding a geranylgeranyl diphosphate synthase may be responsible for the production of the precursor to gibberellin, geranylgeranyl diphosphate (GGDP), while the homologue to the CPS/KS-encoding enzyme is necessary for the cyclization of the final product.

The antiSMASH analysis revealed that the genome of *F. tenuicristatum* has a PKS BGC that is similar to the BGC responsible for radicicol biosynthesis in *Pochonia chlamydosporia* (Figure 6B). Further analysis between these two radicicol BGCs uncovered five orthologous genes, and the organization of this cluster was also conserved. Our study indicates that *PKS116/rdc5* and *PKS13/rdc1* in *F. tenuicristatum*n confer the ability to likely synthesize radicicol as opposed to zearalenone, a secondary metabolite from *F. graminearum* with a similar chemical structure.

A phylogenetic analysis of various NRPS proteins indicated the presence of *NRPS5* and *NRPS9* in *F. bataticola* and *F. euwallaceae* (Figure 3). These two NRPS proteins are known to be involved in the production of the octapeptide fusaoctaxin A in *F. graminearum* [34]. Further analysis between these three homologous BGCs for fusaoctaxin A uncovered conservation in the organization of this cluster, indicating that fusaoctaxin might potentially be produced by *F. bataticola* and *F. euwallaceae*.

## 4. Discussion

Overall, the FSSC isolates included in this analysis revealed that there was a diverse array of potential secondary metabolites that could be synthesized. Collectively, a total of 74 different BGCs (19 PKSs, 2 PKS–PKS hybrids, 21 NRPSs, 1 NRPS–NRPS hybrid, 17 terpene synthases/cyclases, 4 DMATS, 3 phosphonate-producing BGCs, 4 NRPS–PKS hybrids, 1 PKS–terpene hybrid, 1 NRPS–terpene hybrid, and 1 NRPS–DMATS hybrid) were identified in the 10 clade 3 FSSC genomes. The secondary metabolite BGC repertoire for each isolate was unique where no two genomes had the same secondary metabolite biosynthetic potential, even between the two *F. vanettenii* genomes. There were seven PKSs, thirteen NRPSs, two terpene synthases/cyclases, and one DMATS BGC common between all ten FSSC genomes included in this analysis. *PKS3*, *PKS7*, and *PKS8* are frequently found in most *Fusarium* genomes [11,14], and all 10 FSSC isolates in this study carried these BGCs. Conversely, *PKS32*, *PKS33*, and *PKS35* are primarily found in genomes of the FSSC and all 10 FSSC genomes had these BGCs. While the biosynthetic products of PKS32 and PKS33 are not known, PKS35 is responsible for the production of a red pigment [13]. The presence of these PKS BGCs in an unidentified *Fusarium* sp. could aid in their identification as a member of the FSSC; however, it should be noted that *PKS35* has recently been identified in closely related *Fusarium* species complexes such as *F. staphlyeae*, *F. decemcellulare*, and *F. dimerum* [14].

The production of a vast array of secondary metabolites likely contributes to the diverse environmental niche that members of the FSSC can be isolated from. For instance, many naphthoquinones have been isolated from members of the FSSC [3]. PKS3 is involved in the production of naphthoquinones such as fusarubin and oxyjavanicin, and could provide a competitive advantage to the FSSC isolates producing them as napthoquinones have antibacterial activity, especially against Gram-positive bacteria as well as inhibitory activity against protozoa and fungi [9,35].

Members of the FSSC are well-established plant pathogens and collectively have a broad host range, while some within the terminal clade of the FSSC are also associated with clinical infections resulting in fusariosis [3,5,6,7,8]. Some secondary metabolites have been demonstrated to be important virulence factors in fungi other than the FSSC [36]. The NRPS BGCs responsible for the synthesis of siderophores have been documented to be important for virulence for the closely related phytopathogen *F. graminearum* [37]. NRPS6 is conserved in ascomycetes and is responsible for the synthesis of the extracellular siderophore triaceylfusarinine, which is critical for virulence in several plant pathogenic fungi [38]. NRPS1 is responsible for the synthesis of another extracellular siderophore malonichrome, although it appears not to be as important for virulence as NRPS6, while NRPS2 is responsible for the production of the intracellular siderophore ferricrocin. In addition to these three siderophores, another might be produced by NRPS27, which is closely related to NRPS6; this may synthesize metachelins, which have been characterized in *Metarhizium* spp. [39].

In addition to siderophore production, two FSSC genomes included in this study also carried a BGC encoding *NRPS5* and *NRPS9*, which are responsible for fusaotaxin A production in *F. graminearum*. Fusaotaxin A is a virulence factor involved in the cell-to-cell invasion of wheat [34]; however, mutants lacking the cluster display virulence similar to wild-type when inoculated in a maize stalk rot assay, indicating that this secondary metabolite is host-specific [34]. The *NRPS5* homologues in the FSSC isolates are shorter, and therefore, it is hypothesized that they are responsible for producing a smaller product, although the similarity between the *NRPS9* homologues suggests that the initial unit is likely γ–amino butyl acid (GABA), as seen with fusaotaxin A.

Many secondary metabolites have phytotoxic activity and can aid in pathogenicity; in particular, the previously mentioned naphthoquinones are reported to arrest root growth [9]. Another example is radicicol (also known as monorden), which was first identified as a phytotoxin in *F. virguliforme*, a member of clade 2 of the FSSC and is the causative agent of soybean sudden death syndrome (SDS) [40]. Radicicol is capable of producing SDS-like symptoms such as marginal curling and interveinal necrosis on soybean leaves. The secondary metabolite is an inhibitor of heat shock protein 90 [41], and its production in the fungus *Colletotrichum graminicola* is hypothesized to suppress competing microorganisms and the plant defense response [42]. The two core radicicol PKS biosynthetic genes from *F. tenuicristatum* are similar to the *PKS4-* and *PKS13*-containing BGC in *F. graminearum,* which are responsible for the synthesis of the mycotoxin zearalenone [43,44]. A recent genus-wide phylogenetic analysis of PKS proteins from *Fusarium* resolved the radicicol-producing *PKS4* into a separate clade, *PKS116*, and identified structural differences in radicicol and zearalenone that were caused by reducing PKSs and post-PKS modification by other enzymes [14]. While there is similarity between the zearalenone BGC of *F. graminearum* and the radicicol BGC in *F. tenuicristatum*, radicicol biosynthesis could provide a competitive advantage in rhizosphere colonization and/or be directly involved in suppressing the plant host immune response.

The presence of some of the genes from the gibberellin BGC have been previously described in genomes of other *Fusarium* spp. that are more evolutionarily related than members of the FSSC (i.e., *F. mangiferae*, *F. circinatum*, and *F. oxysporum*; [45]). The *F. mori* genome has four of the seven genes in the gibberellin BGC including a homologue of the key *CPS/KS* gene (Figure 6A). While other *Fusarium* genomes have been identified to have intact gibberellin BGCs, these *Fusarium* isolates did not produce gibberellins under standard laboratory conditions [45]. Therefore, it is unlikely that *F. mori* is capable of producing gibberellins and the product of this similar BGC, if any, is unknown.

Several DMATSs and phosphonate BGCs were identified in the FSSC; however, after an analysis of these clusters, none of the final products could be predicted. DMATSs catalyze the prenylation of L-tryptophan to generate dimethylallytrypthohan (DMAT), which is then predicted to be further modified by the accessory proteins also encoded within the cluster [46]. One of the best-known examples of secondary metabolites synthesized by a DMATS encoded in BGCs are the ergot alkaloids of *Claviceps* spp. Phosphonate-producing BGCs are capable of aiding an organism to sequester phosphorus when it is scarce or can function as an antimicrobial metabolite by producing toxic phosphonate compounds [47]. Further experimental characterization of these BGCs is necessary to identify the natural products synthesized and evaluate their biological relevance.

Fungal secondary metabolites have a diverse array of bioactivity as evidenced in the number of compounds that have been developed for clinical use. As a majority of the secondary metabolite BGCs from members of the FSSC produce unknown products, further research into this area could provide alternative therapeutics. Additionally, better knowledge of these compounds could provide alternative options for disease management. Overall, the vast armamentarium utilized by members of the FSSC not only plays a critical role in pathogenesis but likely provides a competitive advantage to these fungi for further expanding their environmental niches.

## Figures and Tables

**Table 1:** List and details of FSSC genomes used in this study.

Species	Strain	Genome Size (Mbp)	Number of Proteins	Source/Reference
*F. vanettenii*	77-13-4	54.59	16,929	[15]
*F. vanettenii*	T23	61.89	18,783	[16]
*F. tenuicristatum*	NRRL 22470	61.51	17,800	[16]
*F. mori*	NRRL 22230	43.64	14,523	[16]
*F. cucurbiticola*	NRRL 22165	42.45	12,147	[16]
*F. bataticola*	NRRL 22400	50.41	16,777	[16]
*F. solani*	FS5	52.93	17,656	[17]
*F. ambrosium*	NRRL 20438	49.04	17,262	NCBI
*F. euwallaceae*	UCR1854	50.55	17,630	NCBI
*F. floridanum*	NRRL 62606	47.42	16,762	NCBI

**Table 2:** Number and type of predicted secondary metabolite producing BGCs in ten clade 3 FSSC genomes.

Species	Strain	Total BGC *^a^*	Total *^b^*					Hybrid BGCs					
			PKS	NRPS	Terpene	DMATS	Phosphonate	PKS–PKS Hybrid	NRPS–NRPS Hybrid	NRPS–PKS Hybrid	PKS–Terpene Hybrid	NRPS–Terpene Hybrid	NRPS–DMATS Hybrid
*Fusarium vanettenii*	77-13-4	39	14	18	6	2	1	1		1			
*Fusarium vanettenii*	T23	42	16	17	8	1	1	1					
*Fusarium tenuicristatum*	NRRL 22470	48	18	18	10	2	2	1		1			
*Fusarium mori*	NRRL 22230	41	15	17	9	1	1	1		1			
*Fusarium cucurbiticola*	NRRL 22165	35	10	18	4	2	1						
*Fusarium bataticola*	NRRL 22400	45	17	22	7	2	1	1	1		1		1
*Fusarium solani*	FS5	43	15	22	6	2	1			2			1
*Fusarium ambrosium*	NRRL 20438	40	14	19	5	2	1			1			
*Fusarium euwallaceae*	UCR1854	40	13	21	5	3	1		1	1		1	
*Fusarium floridanum*	NRRL 62606	38	14	16	6	2	1					1	

*^a^* Total BGC number is the number of BGCs in each genome and can be calculated as = (the sum of core biosynthetic genes from each BGC class—the number of hybrid BGCs). *^b^* The total is the number of the indicated core protein and does not necessarily reflect the number of BGCs with that type of core biosynthetic protein. Some NRPS–PKS hybrids consisted of a single core biosynthetic protein and were classified only under the PKS category. These BGCs were not included in the NRPS–PKS hybrid BGC list.

## Data Availability

Genomes for both *F. vanettenii* genomes, as well as *F. tenuicristatum*, *F. mori*, *F. cucurbuticola*, and *F. bataticola* were obtained from Mycocosm from the Joint Genome Institute (JGI). The genomes of the remaining fungi were obtained from NCBI under Bioproject numbers PRJNA801211 (*F. solani*), PRJNA389173 (*F. ambrosium*), PRJNA341909 (*F. euwallaceae*), and PRJNA389173 (*F. floridanum*).

## Supplementary Materials

The following supporting information can be downloaded at: https://www.mdpi.com/article/10.3390/jof9080799/s1, Figure S1: Phylogenetic analysis of various PKS proteins from FSSC genomes; Figure S2: Genetic organization and conservation of fusarubin/oxyjavanicin BGC across the FSSC genomes and *F. fujikuroi*. Each color represents a different gene and the core biosynthetic gene *PKS3* is indicated; Figure S3: Genetic organization and conservation of the red pigment BGC across the FSSC genomes and *Talaromyces stipitatus*. Each color represents a different gene and the core biosynthetic gene *PKS35* is indicated; Figure S4: Genetic organization and conservation of the malonichrome BGC across the FSSC genomes. Each color represents a different gene and the core biosynthetic gene *NRPS1* is indicated; Figure S5: Genetic organization and conservation of the ferricrocin BGC across the FSSC genomes. Each color represents a different gene and the core biosynthetic gene *NRPS2* is indicated; Figure S6: Genetic organization and conservation of the triaceylfusarinine/fusarinine BGC across the FSSC genomes. Each color represents a different gene and the core biosynthetic gene *NRPS6* is indicated; Figure S7: Genetic organization and conservation of the metachelin BGC across the FSSC genomes and *Metarhizium robertsii*. Each color represents a different gene and the core biosynthetic gene *NRPS27* is indicated; Figure S8: The multi-locus phylogenetic analysis of the terpene GCFs generated by BiG-SCAPE. Only the T-GCFs shared by at least three members of FSSC isolates are shown. T-GCF4, T-GCF5, and T-GCF6 are predicted to produce squalestatin S1, lanosterol/clavaric acid, and aspterric acid, respectively; Figure S9: The multi-locus phylogenetic analysis of the DMATS GCFs generated by BiG-SCAPE. Only the I-GCFs shared by at least three members of FSSC isolates are shown. Figure S10: The multi-locus phylogenetic analysis of the phosphonate GCFs generated by BiG-SCAPE. Only the P-GCFs shared by at least three members of FSSC isolates are shown. Table S1: Secondary metabolite biosynthetic gene clusters (BGCs), their core biosynthetic protein IDs, and the most similar known cluster as predicted by antiSMASH in ten clade 3 FSSC genomes; Table S2: List of reference PKS proteins from various *Fusarium* and fungal species used in this study; Table S3: List of reference NRPS proteins from various *Fusarium* and fungal species used in this study; Table S4: Distribution of polyketide synthases (PKS) identified in ten clade 3 FSSC genomes and their protein IDs; Table S5: Distribution of nonribosomal peptide synthetases (NRPS) identified in ten clade 3 FSSC genomes and their protein IDs; Table S6: Distribution of the terpene synthase/cyclase gene cluster families (T-GCF) identified in ten clade 3 FSSC genomes and their core biosynthetic protein IDs; Table S7: Distribution of the DMATS gene cluster families (I-GCF) identified in ten clade 3 FSSC genomes and their core biosynthetic protein IDs; Table S8: Distribution of the phosphonate-producing gene cluster families (P-GCF) identified in ten clade 3 FSSC genomes and their core biosynthetic protein IDs.
