# Supplementary material for: Inventory of the Secondary Metabolite Biosynthetic Potential of Members within the Terminal Clade of the Fusarium solani Species Complex
Source: J Fungi (Basel). 2023 Jul 28;9(8):799. doi: 10.3390/jof9080799 (PMC10455376; doi:10.3390/jof9080799)
Supplement: Supplementary file 1 [file jof-09-00799-s001.zip › Supplementary_Figures.pdf]

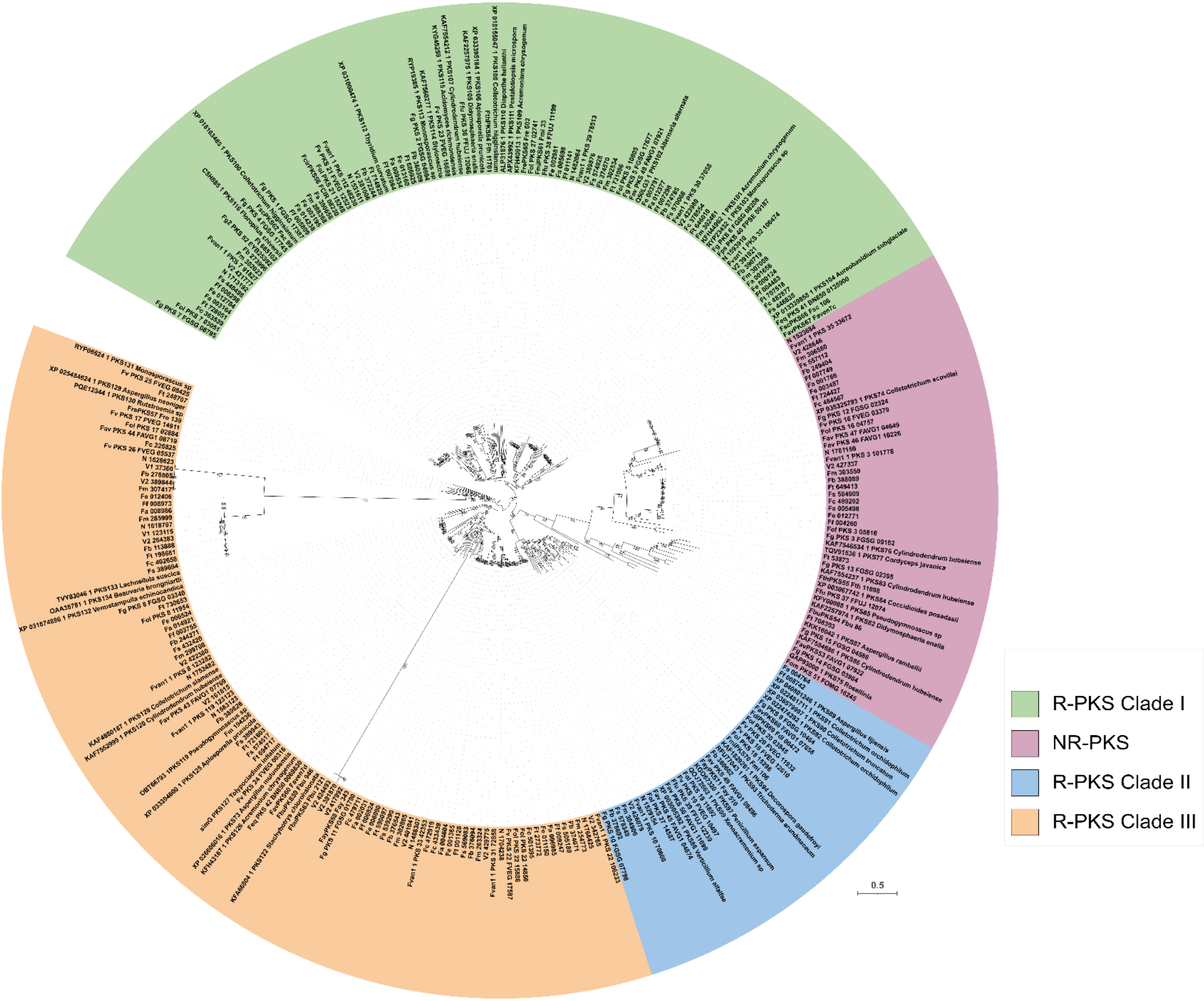

Figure S1. Phylogenetic analysis of various PKS proteins from FSSC genomes.

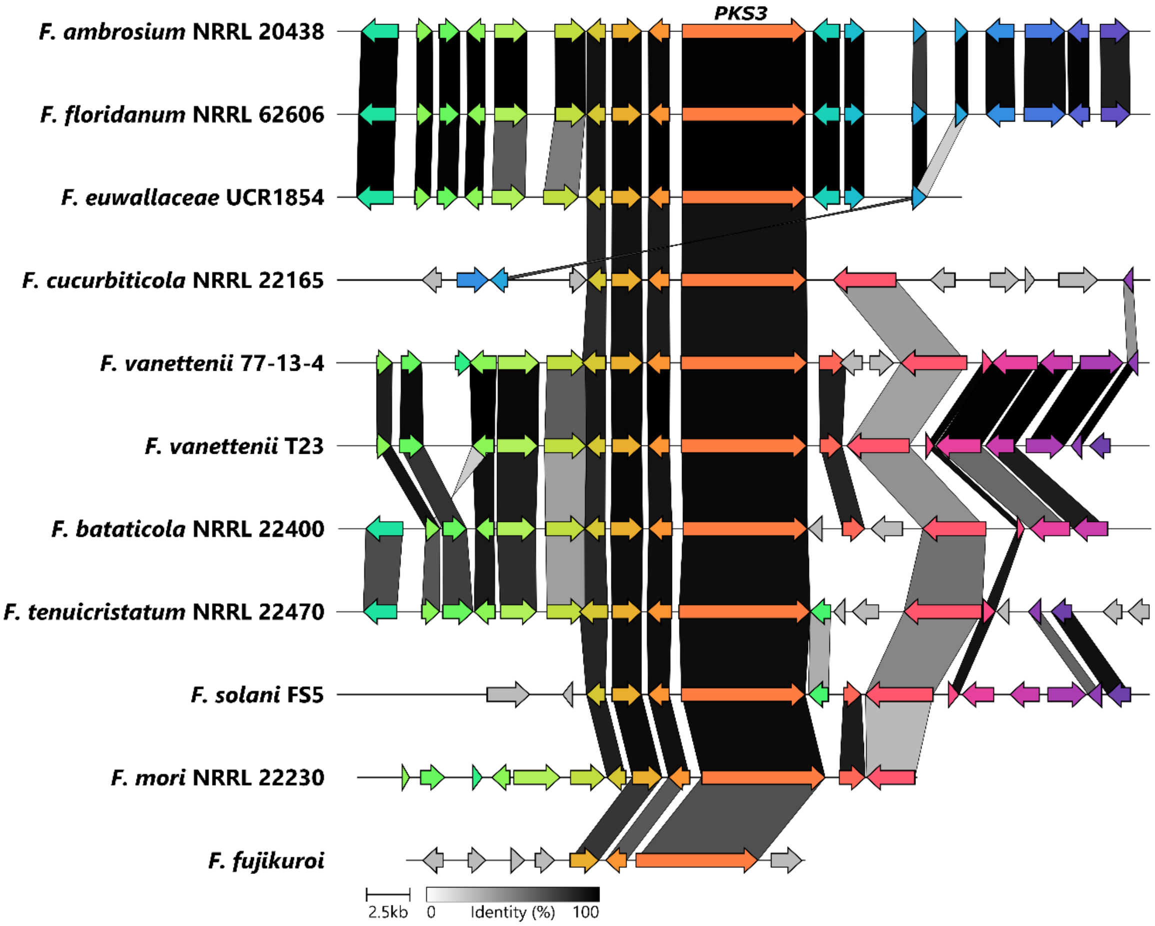

Figure S2. Genetic organization and conservation of fusarubin/oxyjavanicin BGC across the FSSC genomes and *F. fujikuroi*. Each color represents a different gene and the core biosynthetic gene *PKS3* is indicated.

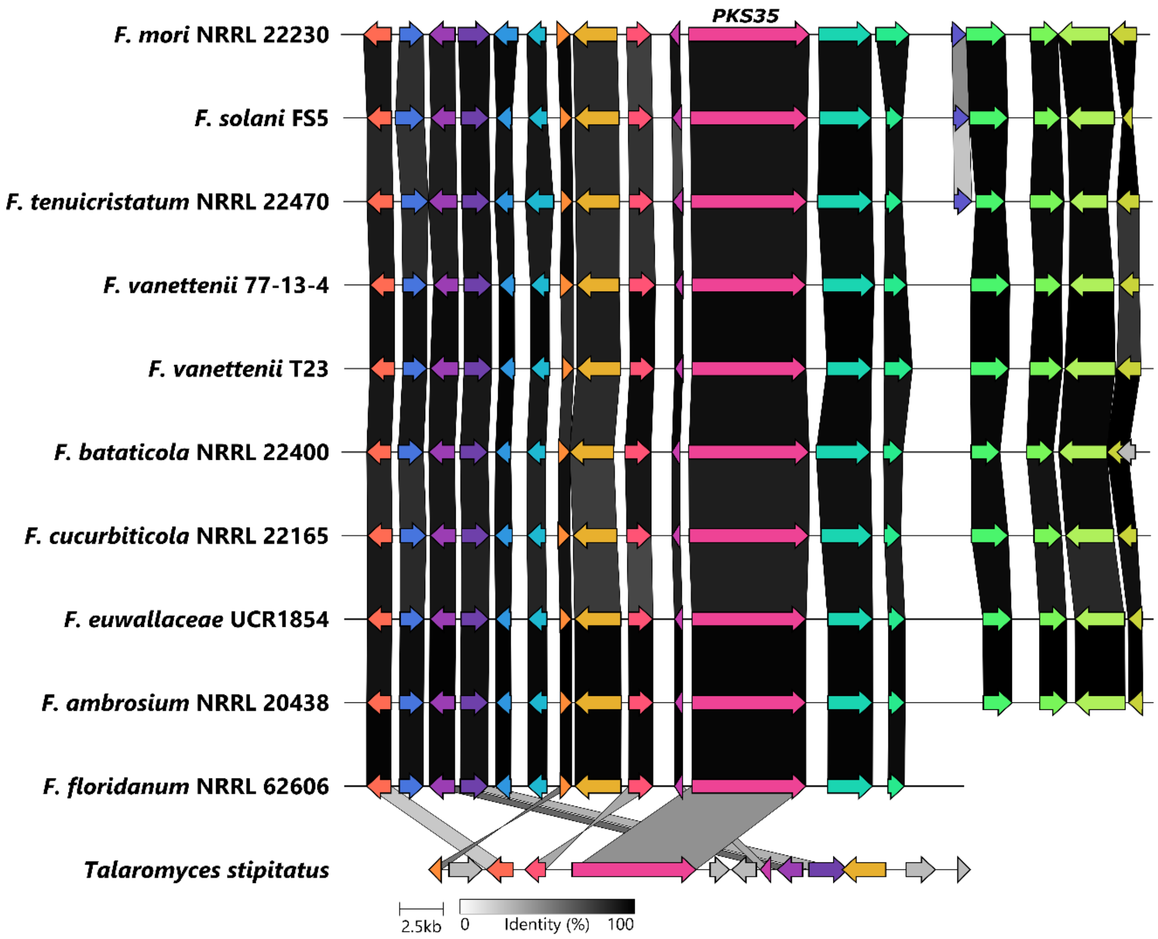

Figure S3. Genetic organization and conservation of the red pigment BGC across the FSSC genomes and *Talaromyces stipitatus*. Each color represents a different gene and the core biosynthetic gene *PKS35* is indicated.

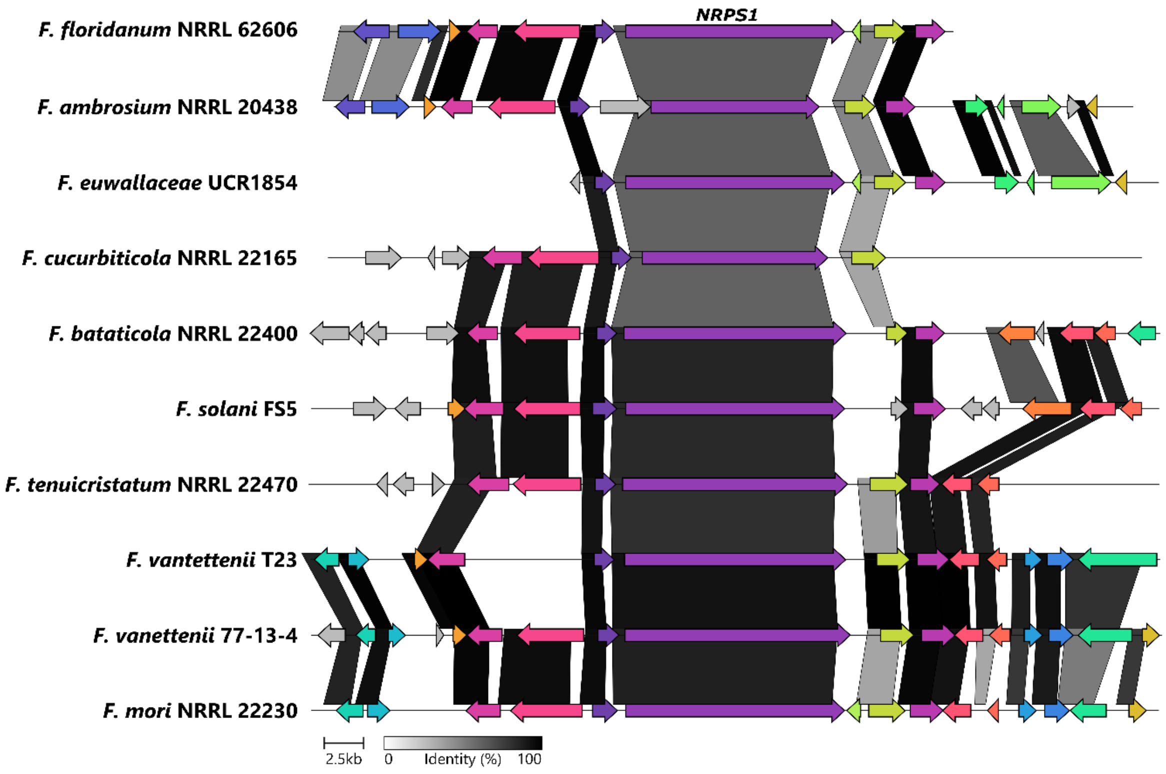

Figure S4. Genetic organization and conservation of the malonichrome BGC across the FSSC genomes. Each color represents a different gene and the core biosynthetic gene *NRPS1* is indicated.

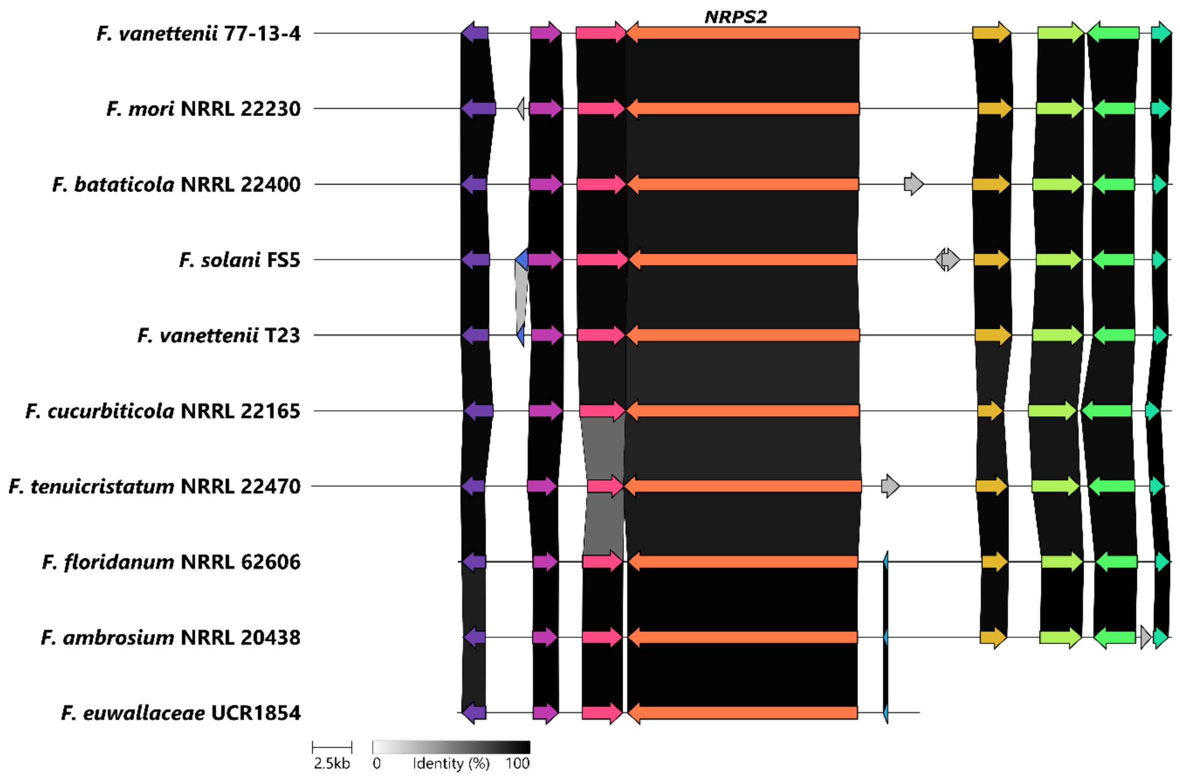

Figure S5. Genetic organization and conservation of the ferricrocin BGC across the FSSC genomes. Each color represents a different gene and the core biosynthetic gene *NRPS2* is indicated.

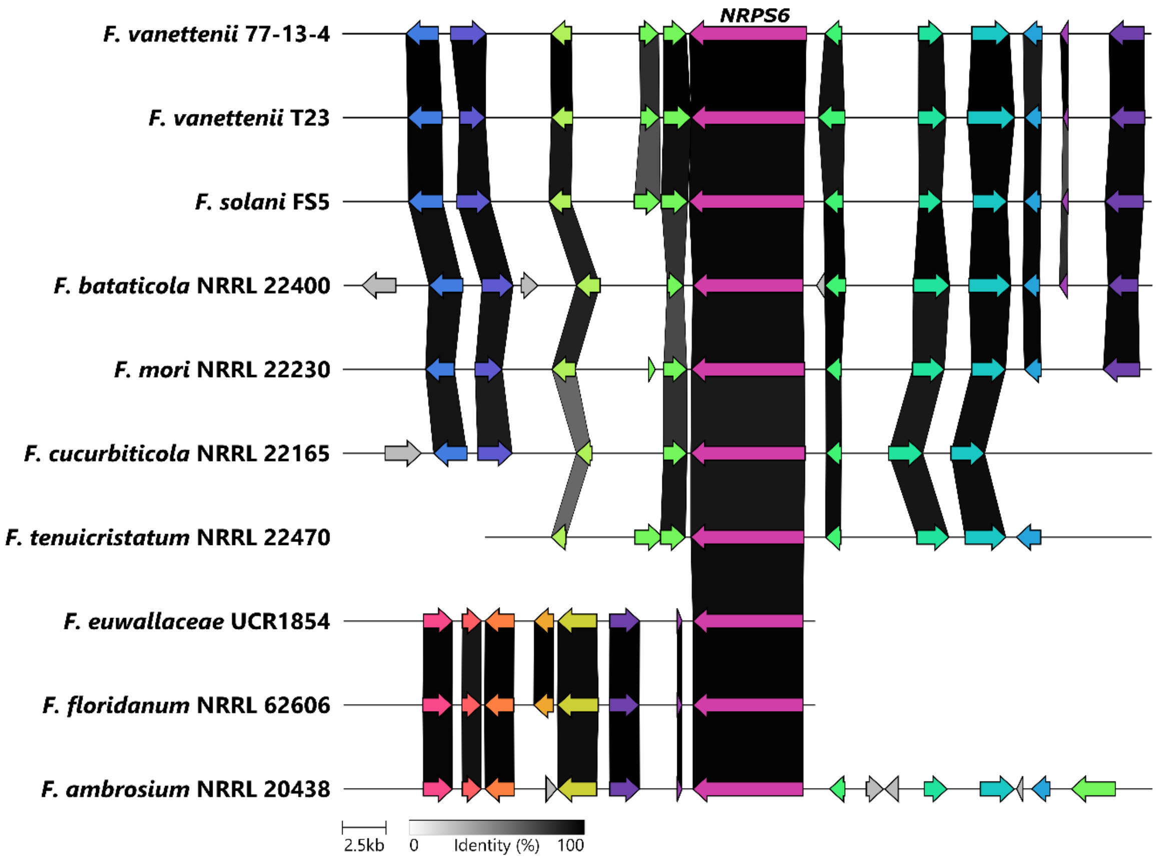

Figure S6. Genetic organization and conservation of the triacetylfusarinine/fusarinine BGC across the FSSC genomes. Each color represents a different gene and the core biosynthetic gene *NRPS6* is indicated.

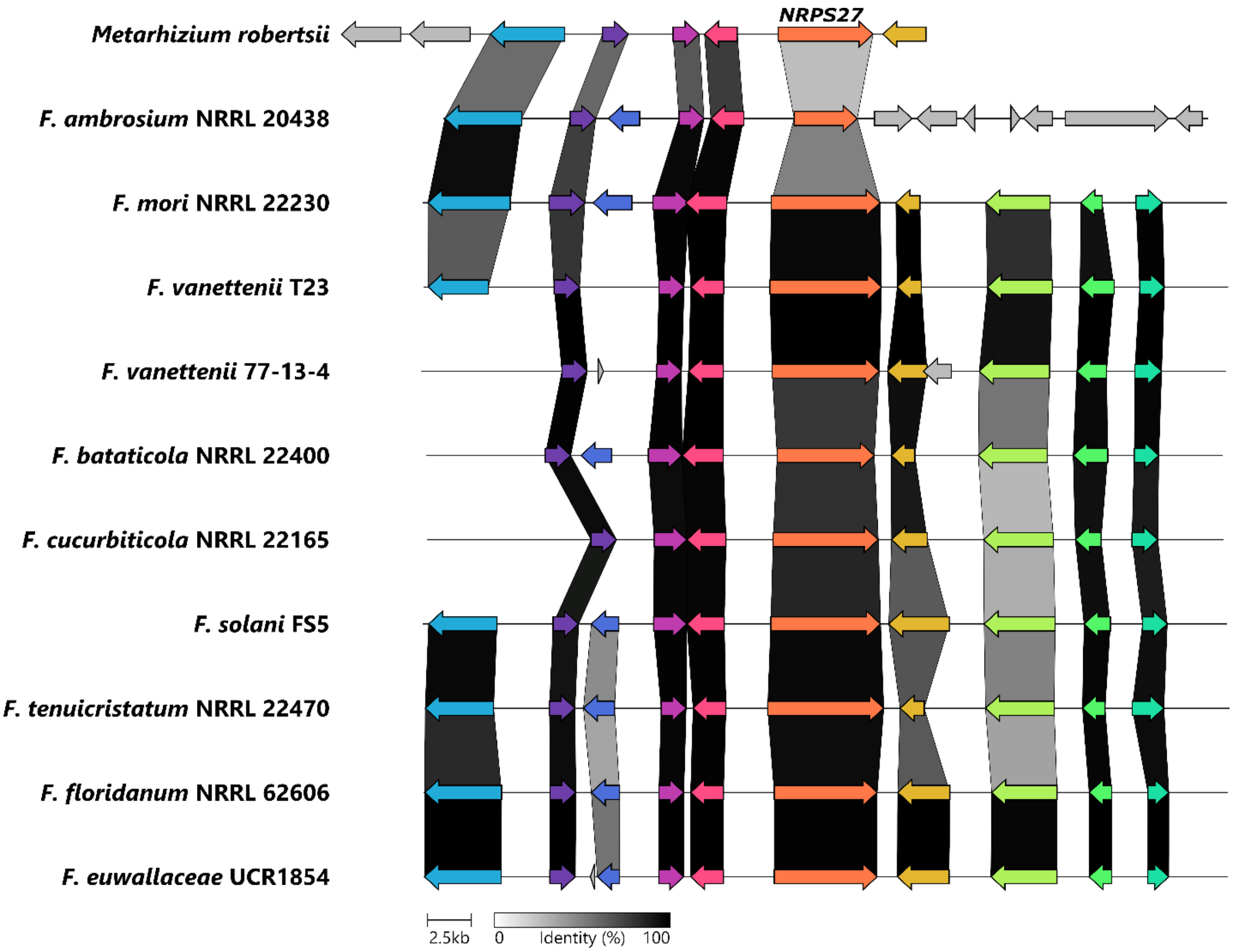

Figure S7. Genetic organization and conservation of the putative metachelin BGC across the FSSC genomes and *Metarhizium robertsii*. Each color represents a different gene and the core biosynthetic gene *NRPS27* is indicated.

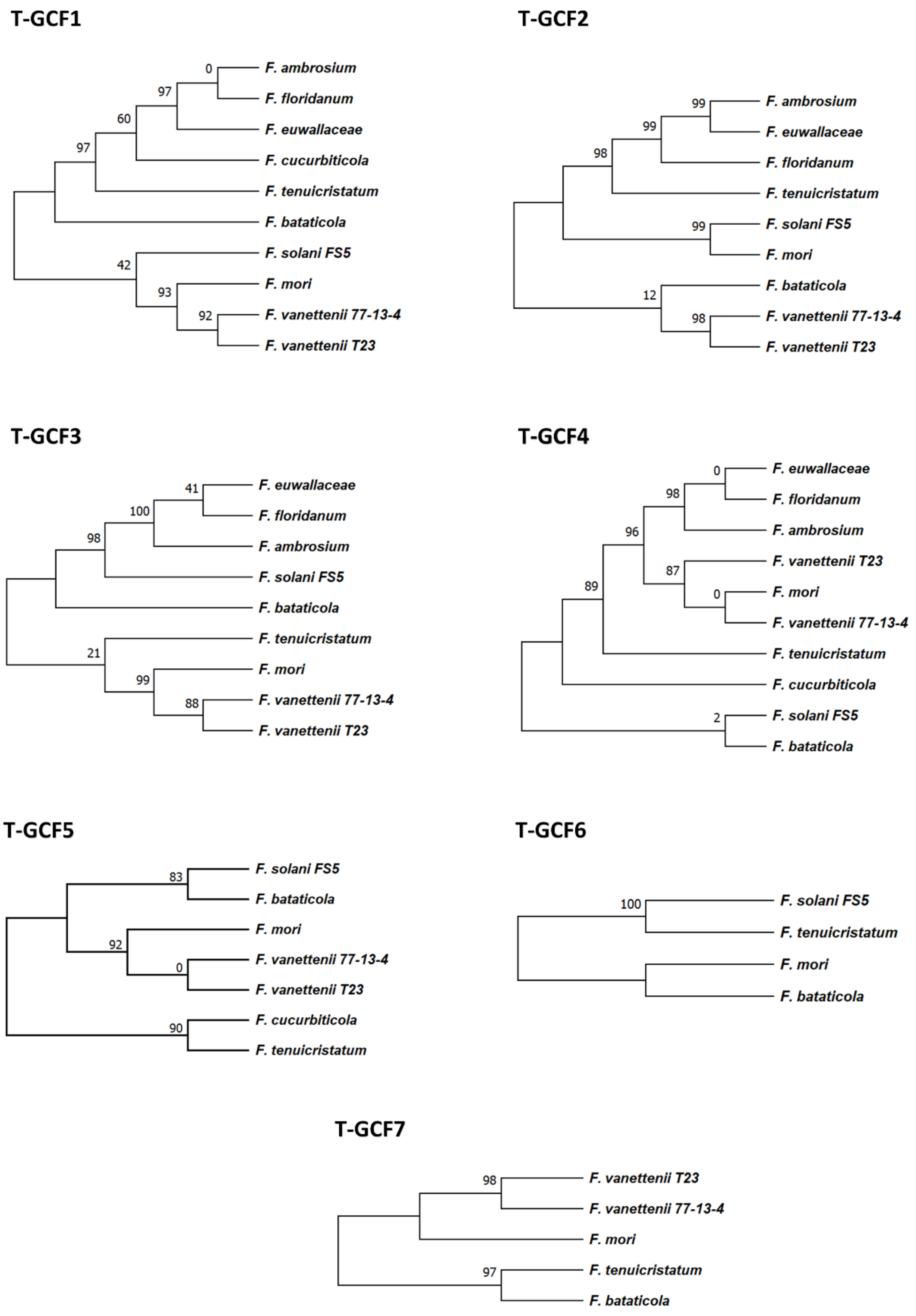

Figure S8. The multi-locus phylogenetic analysis of the terpene GCFs generated by BiG-SCAPE. Only the T-GCFs shared by at least three members of FSSC isolates are shown. T-GCF4, T-GCF5, and T-GCF6 are predicted to produce squalestatin S1, lanosterol/clavaric acid, and aspterric acid, respectively.

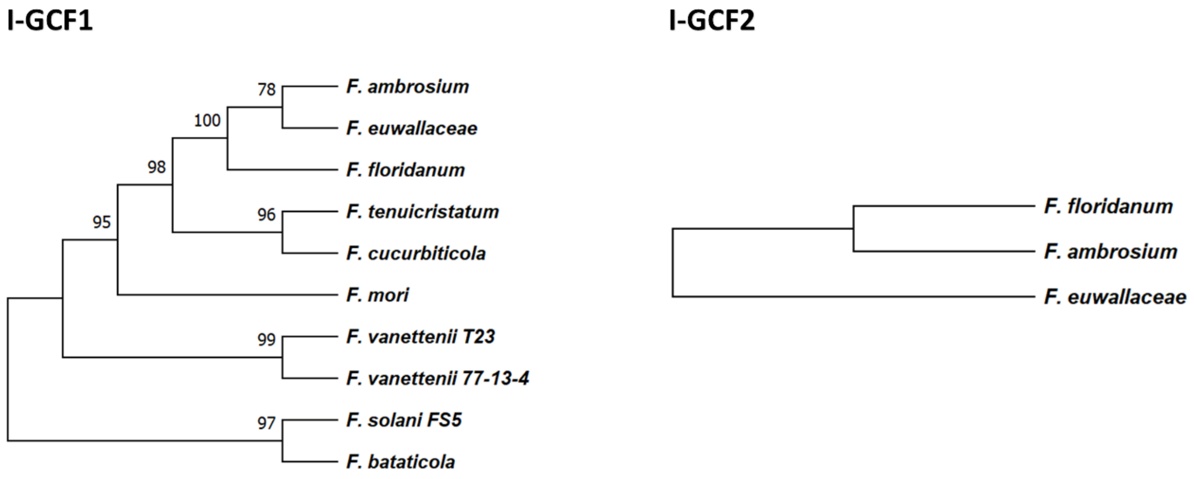

Figure S9. The multi-locus phylogenetic analysis of the indole GCFs generated by BiG-SCAPE. Only the I-GCFs shared by at least three members of FSSC isolates are shown.

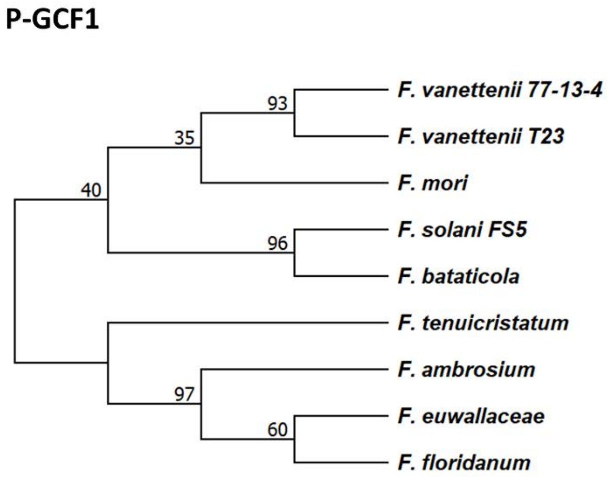

Figure S10. The multi-locus phylogenetic analysis of the phosphonate GCFs generated by BiG-SCAPE. Only the P-GCFs shared by at least three members of FSSC isolates are shown.
